# Melanin production through novel processing of proopiomelanocortin in the extracellular compartment of the auricular skin of C57BL/6 mice after UV-irradiation

**DOI:** 10.1038/srep14579

**Published:** 2015-09-29

**Authors:** Hiroyuki Yamamoto, Tomohiro Yamane, Kazuaki Iguchi, Kiyotaka Tanaka, Arunasiri Iddamalgoda, Keiko Unno, Minoru Hoshino, Atsushi Takeda

**Affiliations:** 1 Department of Microbiology and Molecular Cell Biology, Nihon Pharmaceutical University, 10281 Komuro, Ina-machi, Kitaadachi-gun, Saitama, 362-0806, Japan; 2 Laboratory of Bioorganic Chemistry School of Pharmaceutical Sciences University of Shizuoka, 52-1 Yada, Shizuoka 422-8526, Japan; 3 Department of Research and Development, Ichimaru Pharcos Co. Ltd, 318-1 Asagi, Motosu City, Gifu 501-0475, Japan

## Abstract

The production of melanin is regulated by α-melanocyte-stimulating hormone (α-MSH), which is produced from proopiomelanocortin (POMC). Keratinocytes release POMC along with lower levels of α-MSH and ACTH. To clarify the mechanism of melanogenesis after ultraviolet (UV)-irradiation, this study focused on the expression of POMC and POMC-derived peptides after UV-irradiation. Western blot analysis and immunoassays indicated that both POMC and α-MSH-like immunoreactivity (α-MSH-LI) increased after UV-irradiation. However, other POMC-derived products were very low. In hypophysectomized mice, α-MSH-LI increased to the same level as in control mice after UV-irradiation. Structural analysis revealed that the major α-MSH-LI product was ACTH(1–8). Furthermore, ACTH(1–8) competed with [^125^I]-α-MSH for receptor binding and increased melanin production via a melanocortin-1 receptor. These results suggested that melanin was produced through ACTH(1–8) after UV-irradiation. Trypsin-like enzymatic activity, which is responsible for POMC activation, increased after UV-irradiation and was identified as tryptase. In mast cell-deficient mice, which do not produce tryptase, α-MSH-LI levels were unchanged after UV-irradiation. The present study demonstrates the production of ACTH(1–8) from POMC by tryptase, which is a novel peptide-processing mechanism in the extracellular compartment of the skin.

UV-irradiation induces skin damage of many forms: photoaging^1^, DNA photodamage^2^, inflammation^2^,^3^, tanning^3^ and skin cancer^4^. Pigmentation is a protective response of the skin against these forms of UV-mediated damage, where melanin is produced in melanocytes in the epidermis after UV-irradiation to the skin^5^. Melanin is an antioxidant and free radical scavenger^6^. Melanin production induced by UV-irradiation is regulated by several intermediary factors, such as growth factors, bioactive peptides and lipid mediators^7^. α-Melanocyte-stimulating hormone (α-MSH) and its intermediate form, adrenocorticotropic hormone (ACTH), are major bioactive peptides involved in melanogenesis^8^. α-MSH and ACTH are produced from the proopiomelanocortin (POMC) precursor by prohormone convertase (PC) 1/3 and PC2 in the secretary vesicles in the pituitary and hypothalamus^9^,^10^.

α-MSH and ACTH are released from the pituitary and can be detectable in blood circulation^11^. However, in adult humans, the pituitary lacks an intermediate lobe, so α-MSH is undetectable in circulation. It has been reported that these peptides, when originating from the pituitary, have an effect on skin pigmentation in human^12^. However, skin pigmentation is preferentially induced in areas irradiated with UV through an unknown induction mechanism. It is possible that skin pigmentation shows induced local responses to UV in irradiated areas.

Conversely, POMC-derived peptides such as ACTH, α-MSH and ß-endorphin (ß-END) are also found in melanocytes, fibroblasts, keratinocytes, and in mammalian skin biopsies^13^,^14^,^15^,^16^,^17^,^18^,^19^,^20^,^21^,^22^. Recently, Rousseau *et al.* reported that human keratinocytes and melanocytes primarily release POMC in response to stimulation with corticotrophin-releasing hormone (CRH), while releasing α-MSH and ACTH at low levels^23^.

The type 1 melanocortin receptor (MC-1R), expressed in melanocytes, contributes to melanin production^24^. α-MSH and ACTH are known agonists for MC1-R but the affinity of POMC for MC-1R is markedly low^23^. To clarify the mechanism of melanin production after UV-irradiation, we assessed the expression of POMC and POMC-derived peptides after UV-irradiation to the skin of the murine auricle. The present paper investigates melanin production through the novel processing of POMC in the skin’s extracellular compartment after UV-irradiation.

## Results

### α-MSH-like immunoreactivity is detectable in UV-irradiated auricular tissue

**Figure 1:**
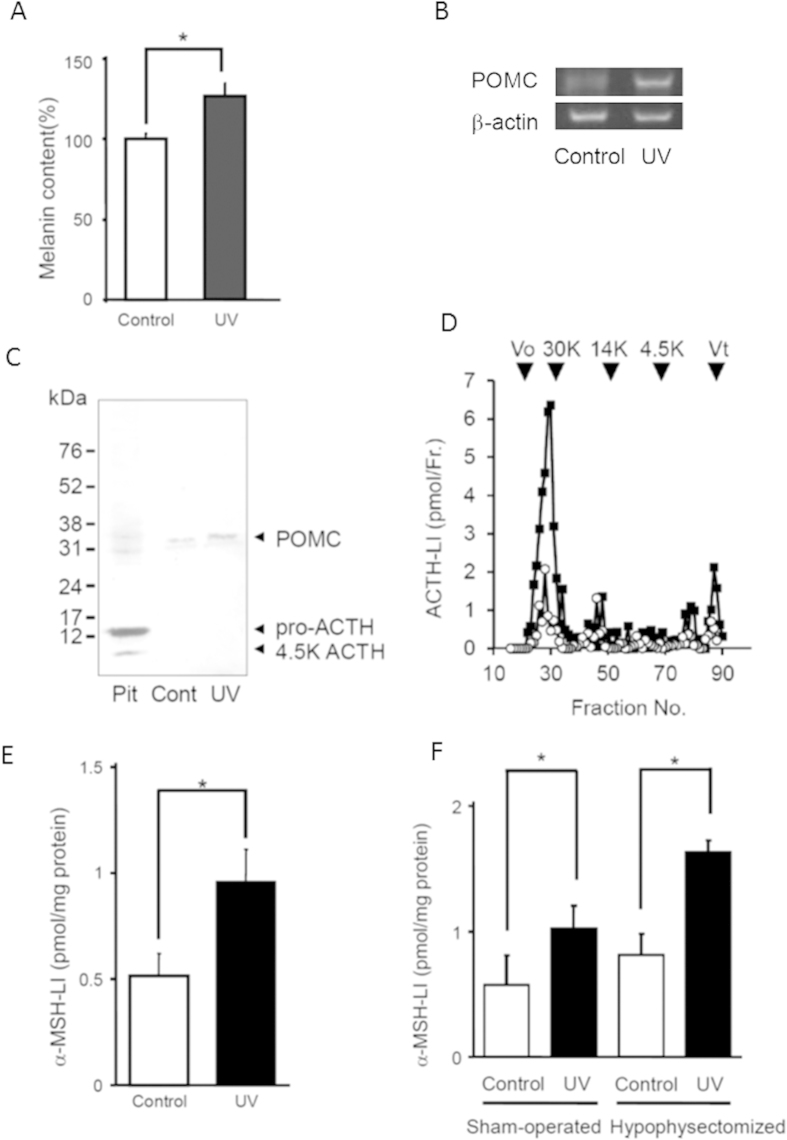
Effects of UV-irradiation on mouse auricular skin. (**A**). Melanin content in the auricles was determined by spectrometry at 475  m. Each bar and line represents the mean ± SEM (n = 4–8, *p < 0.05 vs. control). (**B**). RT-PCR for POMC mRNA and β-actin mRNA (control) in the auricles. (**C**). Western blots of ACTH-LI using antibody R22 against ACTH. Numbers indicate the molecular weights of marker proteins. ‘Pit’ denotes pituitary; ‘Cont’, non-irradiated auricle (control); ‘UV’, UV-irradiated auricle. (**D**). Auricles after UV-irradiation were subjected to gel filtration chromatography to determine the fractions of the ACTH-LI. Filled squares indicate the elution pattern of ACTH in UV-irradiated auricles and open circles indicate that of ACTH in control auricles. Arrows indicate molecular markers (30 K, carbonic anhydrase: 14 K, lysozyme: 4.5 K, ACTH). Vo and Vt indicate void volume (blue dextran) and bed volume (dibutyryl cAMP), respectively. (**E**). α-MSH-LI in the auricles after UV-irradiation. Each bar and line represents the mean ± SEM (n = 4, *p < 0.05 vs. control). (**F**). Expression of α-MSH-LI in the auricles from hypophysectomized mice and its increase after UV-irradiation. Each bar and line represents the mean ± SEM (n = 4, *p < 0.05 vs. control).

To examine the mechanisms driving skin pigmentation following *in vivo* UV-irradiation, we exposed the right auricles of mice to UV light and kept left auricles as controls with no UV exposure. Skin melanin level increased significantly in UV-irradiated auricles [Fig. 1A]. Semi-quantitative RT-PCR, Western blot analysis, and gel filtration chromatography revealed that the expression of POMC also increased in UV-irradiated auricles [Fig. 1B–D]. Conversely, ACTH and pro-ACTH were detected at low levels in auricle extracts with or without UV-irradiation. In contrast, the pituitary abundantly contained pro-ACTH (13 kDa) and ACTH (4.5 kDa), in addition to POMC (32 kDa).

Moreover, α-MSH-specific radioimmunoassay (RIA) revealed that α-MSH-LI increased significantly in UV-irradiated auricles [Fig. 1E]. Therefore, we measured α-MSH-LI in UV-irradiated auricles of hypophysectomized mice to determine whether α-MSH-LI was produced locally in UV-irradiated auricles. α-MSH-LI increased significantly, similar to sham-operated mice [Fig. 1F].

### Structural analysis of peptides producing α-MSH-like immunoreactivity

**Figure 2:**
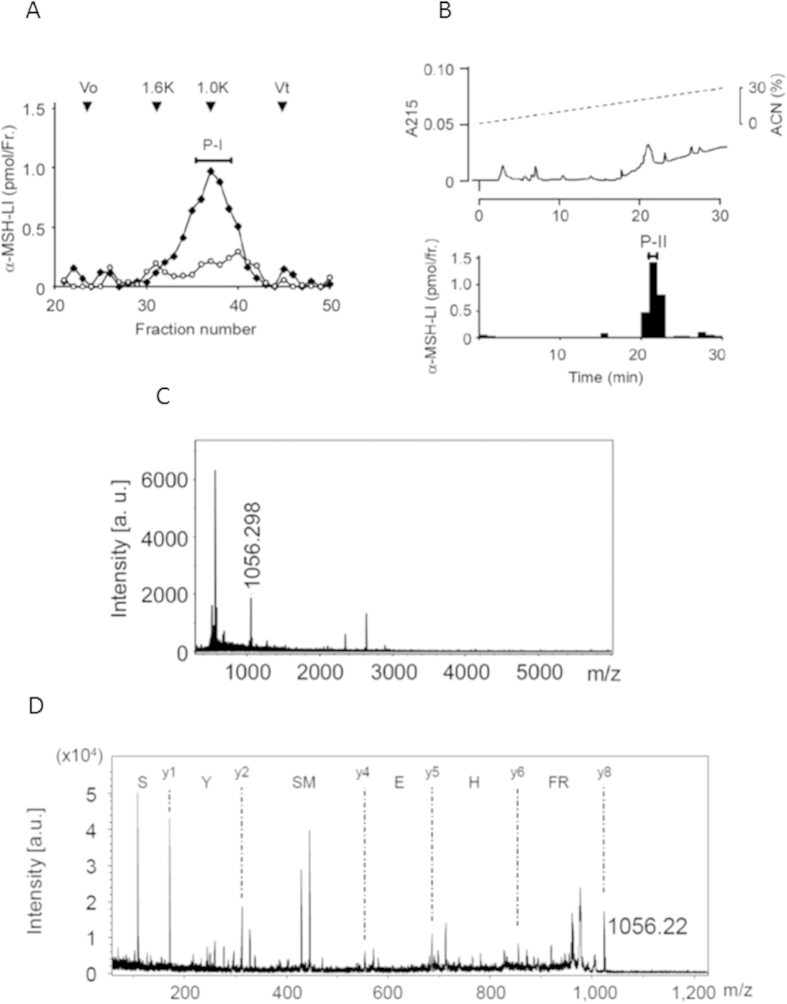
Structural analysis of α-MSH-LI in the auricles. (**A**). Auricles after UV-irradiation were subjected to gel filtration chromatography to determine the fractions of α-MSH-LI. Filled squares indicate the elution pattern of α-MSH-LI in UV-irradiated auricles and open circles indicate that of α-MSH-LI in control auricles. Arrows indicate molecular markers (1.6 K, α-MSH: 1.0 K, ACTH(1–8)). The major α-MSH-LI was collected as the P-I fraction. Vo and Vt indicate void volume (blue dextran) and bed volume (dibutyryl cAMP), respectively. (**B**). The P-I fraction was subjected to reverse-phase HPLC to determine the fraction of α-MSH-LI. Most α-MSH-LI was collected as the P-II fraction. The P-II fraction was analyzed using MALDI-TOF MS (**C)** and MS/MS (**D**). The peak at m/z 1056 was identified as ACTH(1–8), as determined by MS/MS.

We measured the molecular weights of peptides producing α-MSH-LI in the auricle using gel filtration chromatography. The gel filtration profile of α-MSH-LI in control auricles peaked at about 1.6 kDa, which we estimated as α-MSH. The α-MSH-LI in UV-irradiated auricles showed a main peak at about 1.1 kDa, while the 1.6 kDa peak was apparently undetected in UV-irradiated auricles [Fig. 2A]. Pro-ACTH and ACTH, which are eluted in the void volume, were undetectable in auricles with or without UV-irradiation, in agreement with the Western blotting results.

The 1.1 kDa α-MSH-LI peak was then analyzed by reverse-phase chromatography [Fig. 2B]. The α-MSH-LI was eluted as one peak and identified as ACTH(1–8) based on MALDI-TOF MS [Fig. 2C] and MS/MS data [Fig. 2D]. In contrast, acetylated α-MSH-related peptides were not detected in UV-irradiated auricles. These results indicated that ACTH(1–8) was produced by cleaving the Arg and Lys residues of POMC, pro-ACTH or ACTH, reflecting the action of a trypsin-like protease.

### Effects of ACTH(1–8) on melanogenesis and MC-1R binding

**Figure 3:**
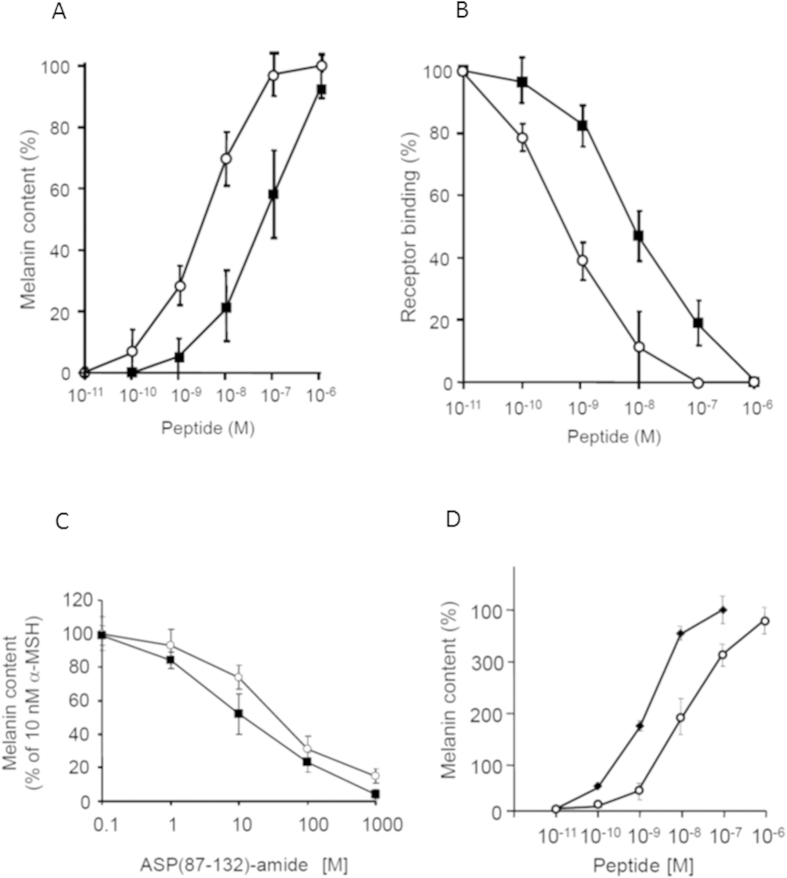
ACTH(1–8)-mediated melanin production via the MC-1R. (**A**). The Radioreceptor assay of ACTH(1–8) (open circle) and α-MSH (closed circle) in mouse melanoma B16 cells. B16 mouse melanoma cells were incubated with [^125^I]-α-MSH and 10^−7^–10^−11 ^M ACTH(1–8) or 10^−6^–10^−11 ^M α-MSH. The receptor binding of [^125^I]-α-MSH was expressed as 100% in the absence of α-MSH and 0% in the presence of 1 μM α-MSH and calculated as relative activity. (**B**). ACTH(1–8)- and α-MSH-mediated melanin production. B16 mouse melanoma cells were incubated with 10^−7^–10^−11^ M ACTH(1–8) (open circle) or 10^−6^–10^−11 ^M α-MSH (closed circle). Melanin levels are expressed as 100% and 0% in the presence of 1 μM α-MSH and absence of α-MSH, respectively, and calculated as the relative activity. C. Dose-dependent effect of ASP(87–132)-amide, an MC1R antagonist, inhibiting melanin production induced by 10^−8 ^M α-MSH (open circle) or 10^−7 ^M ACTH(1–8) (closed circle). Each point and line represents the mean ± SEM (n = 4). D. ACTH(1–8)- and α-MSH-mediated melanin production on human melanocytes. Human melanocytes were incubated with 10^−7^–10^−11 ^M ACTH(1–8) (open circle) or 10^−6^–10^−11 ^M α-MSH (closed circle). Melanin levels are expressed as 100% and 0% in the presence of 1 μM α-MSH and absence of α-MSH, respectively, and calculated as the relative activity.

The binding of ACTH(1–8) to MC-1R was tested in mouse melanoma B16 cells. In the radioligand binding assay using [^125^I]-α-MSH [Fig. 3A], the receptor binding of ACTH(1–8) (Kd = 88 nM) was approximately 30 times less than that of α-MSH (Kd = 3.1 nM). To examine the effect of ACTH(1–8) on melanin production, B16 cells were incubated with 10^−7^–10^−11^ M ACTH(1–8). We increased melanin levels dose-dependently, as well as for α-MSH. At the maximal dose of 1000 nM, the melanin production with ACTH(1–8) (EC_50 _= 80 nM) was approximately 20 times less than that with α-MSH (EC_50 _= 4.3 nM) [Fig. 3B]. Furthermore, melanin production with ACTH(1–8) was competitively attenuated in the presence of ASP(87–132)-NH_2_, a MC-1R antagonist, as well as with α-MSH [Fig. 3C], indicating that ACTH(1–8) activates MC-1R to elicit melanogenesis. Moreover, we assessed the effect of ACTH(1–8) on melanogenesis using human melanocytes. ACTH(1–8) potently induced similar levels of melanogenesis in human melanocytes and B16 melanoma cells (Fig. 3D).

### Expression of a trypsin-like protease in response to UV-irradiation

**Figure 4:**
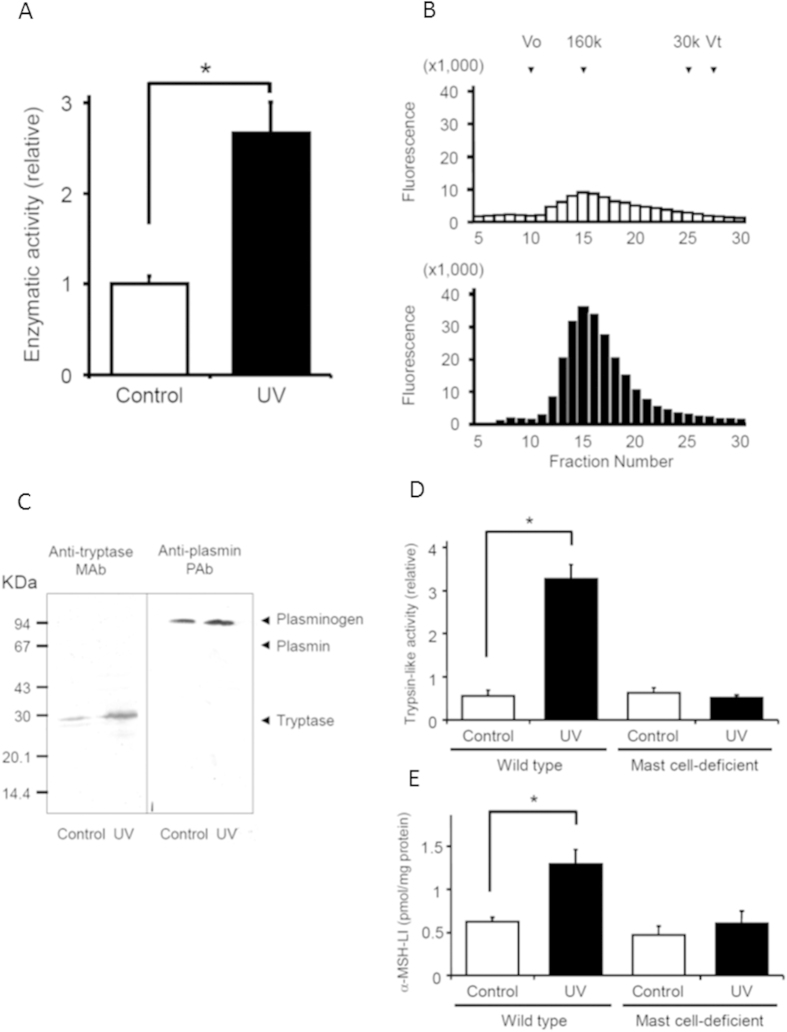
Lack of increase in tryptase activity and α-MSH-LI in the auricles of mast cell-deficient mice after UV-irradiation. (**A**). The activity of trypsin-like protease in the auricles after UV-irradiation. The trypsin-like protease was identified using a Boc-Phe-Ser-Arg-MCA cleavage assay. Each bar and line represents the mean ± SEM (n = 4, *p < 0.05 vs. control). (**B**). After UV-irradiation the auricles were subjected to gel filtration chromatography to determine any trypsin-like protease activity. Arrows indicate molecular markers (160 K, aldolase: 30 K, carbonic anhydrase). Vo and Vt denote void volume (blue dextran) and bed volume (dibutyryl cAMP), respectively. (**C**). Western blotting analysis of tryptase and plasmin in the auricles after UV-irradiation. D and E. Changes in trypsin-like protease activity (**D**) and α-MSH-LI (**E**) in auricles of mast cell-deficient (WBB6F1-W/Wv) and wild-type mice (WBB6F1-+/+) after UV-irradiation. Each bar and line represents the mean ± SEM (n = 4, *p < 0.05 vs. control).

Trypsin-like protease activity was measured using a Boc-Phe-Ser-Arg-MCA cleavage assay. Trypsin-like protease activity was elevated in UV-irradiated auricles [Fig. 4A]. The molecular weight of the trypsin-like protease was measured using gel filtration and Western blot analysis. The major fraction was a molecular mass of about 160 kDa after gel filtration [Fig. 4B]. Western blot analysis revealed the presence of tryptase, a trypsin-like protease, with a molecular mass of about 30 kDa [Fig 4C]. Tryptase forms a tetramer protein with a molecular mass of about 160 kDa, suggesting that tryptase is a major trypsin-like protease involved in POMC processing in UV-irradiated skin.

### Response after UV-irradiation to the auricles in mast cell-deficient mice

To assess the role of tryptase in POMC processing, which is expressed in mast cells, we measured trypsin-like protease activity in auricles irradiated with UV. Trypsin-like protease activity increased markedly in UV-irradiated auricles of wild-type (WBB6F1-+/+) mice, but not in UV-irradiated auricles of mast cell-deficient (WBB6F1-W/Wv) mice. The α-MSH-LI also increased in UV-irradiated auricles of wild-type mice, but not in mast cell-deficient mice [Fig. 4E].

## Discussion

Melanin plays an important role in protecting skin after UV-irradiation. α-MSH is a major catalyst for melanogenesis in the skin following UV-irradiation. α-MSH-LI and ACTH-LI may be present in culture media of normal keratinocytes, fibroblasts, melanocytes, and their cell extracts^13^,^14^,^15^,^16^,^17^,^18^,^19^,^20^,^21^,^22^. Although POMC and POMC-derived peptides such as ACTH and α-MSH are released from the intermedia of the pituitary, it is unlikely that pituitary secretions contribute to local pigmentation. In the present report, POMC-related peptides were detected in the UV-irradiated auricles of hypophysectomized mice. These results suggest that skin regulates its own pigmentation. Research by Slominski *et al.* supports this idea, revealing that keratinocytes and melanocytes released POMC-related peptides following stimulation by corticotrophin-releasing hormone (CRH)^25^. In addition, keratinocytes and melanocytes release large amounts of POMC compared to α-MSH and ACTH^23^. However, POMC only slightly influenced melanogenesis via MC-1R^23^. Recent studies showed small lung carcinoma cells can release neuropeptide progalanin that are activated in the extracellular compartment^26^,^27^ and contribute to angiogenesis^28^. In the present study, we explored the idea that POMC released from keratinocytes is activated in the extracellular compartments of skin and involved in melanogenesis in the auricles after exposure to UV.

**Figure 5:**
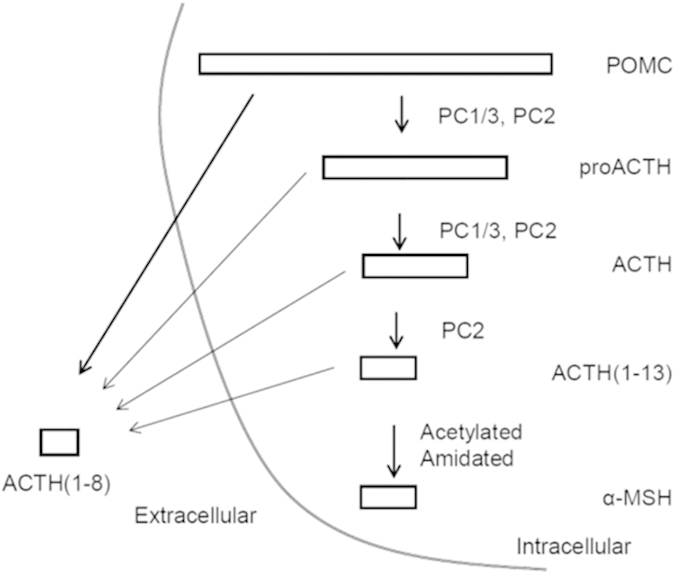
Processing of proopiomelanocortin in the skin. POMC: proopiomelanocortin, PC: prohormone convertase, α-MSH: α-melanocyte-stimulating hormone.

Western blot analysis, gel filtration chromatography and immunoassay indicated that both POMC production and α-MSH-LI increased in UV-irradiated auricles. However, other POMC-derived products, such as pro-ACTH and ACTH, were detected, but at very low levels, suggesting the processing mechanism of POMC varies between the pituitary and skin. α-MSH-LI can be present in blood and affect peripheral tissue^29^. Conversely, α-MSH-LI expression increases in UV-irradiated skin^30^, which may contribute to melanogenesis in UV-irradiated skin. We used hypophysectomized mice to determine whether α-MSH-LI is induced in the skin independent of the pituitary. α-MSH-LI was detected at almost similar levels between hypophysectomized and control mice, and increased at similar rates after UV-irradiation. Structure analysis revealed that the major component of α-MSH-LI was ACTH(1–8). Gel filtration analysis indicated that α-MSH and ACTH levels, which did not show significant peaks, showed no change in UV-irradiated skin. Furthermore, synthetic ACTH(1–8) competed with [^125^I]-α-MSH for receptor binding and increased melanin production. These results suggest that ACTH(1–8) produces melanin in the skin after UV-irradiation. Further, α-MSH is acetylated by N-acetyl transferase in the secretary vesicles of the pituitary. Slominski *et al.* characterized POMC-related peptides in human skin using liquid chromatography-mass spectrometry (LC-MS)^25^. The reports showed that POMC was processed by prohormone convertase 1/3 and 2 to the des-acetylated forms ACTH and ACTH(1–13), and acetylated-amidated form α-MSH. In this paper, we characterized a shorter form of ACTH(1–8) found in the UV-irradiated auricle. Interestingly, we cannot detect acetylated ACTH(1–8). Because neuropeptides including α-MSH are acetylated by N-acetyl transferase in the secretary vesicles, we suggest POMC or ACTH, and not α-MSH, processed ACTH(1–8) (Fig. 5). In addition, because POMC was found in larger amounts than ACTH or α-MSH, POMC was considered the major source of ACTH(1–8). Combined, these results suggest that POMC is uniquely processed for melanogenesis in the skin after UV-irradiation.

Structure-activity analyses of α-MSH have reported information on the essential sequence required for MC-1R binding and melanin production^31^,^32^,^33^. Bioassay studies on melanin production have identified the peptide Ac-His-Phe-Arg-Trp-NH_2_ (corresponding to α-MSH(6–9)) as the minimal synthetic fragment capable of eliciting a melanotropic response. However, Ac-His-Phe-Arg-Trp-NH_2_ had 400,000-fold less activity (EC_50_ 20 μM) than α-MSH in a frog skin bioassay^31^ and approximately 7,000-fold less activity (EC_50_ 1 μM) in a lizard skin bioassay^32^. Furthermore, the N-terminal region of α-MSH is important in MC-1R binding^31^. In the mouse melanoma, ACTH(1–8) [EC_50_ 80 nM] showed 20–30-fold less activity in melanin production than α-MSH, but the potency against melanogenesis of ACTH(1–8) was about equal. ASP(87–132)-NH_2_, a MC-1R antagonist, decreased both ACTH(1–8)- and α-MSH-mediated melanogenesis, suggesting the N-terminal sequence of α-MSH plays an important role in melanin production through MC-1R activation. POMC also produces γ-MSH, which increases melanin production and is potent when binding to MC-1R, but at low potency levels similar to ACTH(1–8)^34^. Studies show γ-MSH acts as a competitive antagonist against MC-1R^34^, so ACTH(1–8) may play a similar role. These conclusions indicate that the activity of ACTH(1–8) was both agonistic and antagonistic under a variable environment.

C57BL/6 mice are well-studied models for exploring pigment mechanisms and expression of POMC and POMC-related peptides. ACTH, α-MSH, β-endorphin and β-MSH, all derived from POMC, as well as a corticotropin-releasing hormone receptor, were detected during a hair cycle^35^,^36^,^37^,^38^. Interestingly, the expressions of these peptides were regulated in space and time for each hair cycle stage. Additionally, corticotropin-releasing hormone regulated the expression of these POMC-related peptides in a similar manner to hypothalamus-pituitary regulation. In this study, UV-irradiation induced the expression of tryptase released from mast cells, resulting in ACTH(1–8) production from POMC and ACTH-related peptides. Melanin was produced in two forms, pheomelanin (yellow /red) and eumelanin (black). In the POMC-deficient C57BL/6 mice study, melanocytes continuously produced eumelanin and α-MSH caused a shift from producing pheomelanin to eumelanin^39^. These results show that α-MSH affected not only melanogenesis, but the level of eumelanin changed the color tone. In the present study, UV-irradiation produced black auricular skin in the C57BL/6 mice, supported by a previous report^39^.

Trypsin-like enzymatic activity increases after UV-irradiation^40^. Tryptase is a major serine protease present in mast cell secretary vesicles. Previous studies report tryptase expression increases in normal human skin after UV-irradiation^40^,^41^. In the present study, Western blot analysis identified tryptase as the major trypsin-like enzyme responsible for processing POMC. In mast cell-deficient mice, which do not produce tryptase, trypsin-like protease activity and the α-MSH-LI were unchanged in the auricle after UV-irradiation, unlike the increases seen in wild-type mice. In UV-irradiated skin, tryptase contributes to the pigmentation process via protease-activated receptor-2 (PAR-2) activation. PAR-2 activation induces melanosome transfer by increasing the phagocytosis of melanosomes by keratinocytes^42^.

## Conclusions

The present study demonstrates the local production of ACTH(1–8) from POMC and POMC-related peptides by tryptase, which is a novel peptide-processing mechanism in the skin’s extracellular compartment. This process may play an important role in the pigmentation of skin following UV irradiation.

## Experimental procedures

### Experimental animals

Male C57BL/6 mice, mast cell-deficient male WBB6F1-W/Wv mice (8 weeks old), and non-deficient male WBB6F1-+/+ mice (8 weeks old) were purchased from Japan SLC, Inc. Prior to experiments, mice were housed under standard laboratory conditions (23 ± 1 °C, 55 ± 5% humidity) and had access to tap water and food *ad libitum*. Lights were automatically turned on at 8:00 and off at 20:00. C57BL/6 mice were hypophysectomized through the external ear canal. Successful removal of the pituitary gland was confirmed by post-experimental craniotomy.

### Sample preparation and measurement of melanin content

The right auricle was exposed to UV-irradiation at 500 mJ cm^−2^ using a 11FL-40SE lamp (Toshiba, Tokyo, Japan). Twenty-four hours after UV-irradiation, the auricle was removed and immediately boiled in 5 ml of 0.1 M acetic acid for 10 minutes. The auricles were cooled in an ice bath, homogenized in 0.1 M acetic acid, and centrifuged at 10,000 rpm for 30 minutes. The supernatants were stored at −80 °C for RIA or Western blot analysis. The pellets were dissolved in 1 M NaOH at 60 °C, and melanin content was measured by a spectrophotometer at 475 nm. The pituitary extracts for Western blotting were prepared according to the same method as the auricle samples.

### Radioimmunoassay

Radioimmunoassay (RIA) was performed with antibody R24 and R22, which were raised in rabbits against synthetic diAc-α-MSH and ACTH, respectively. The α-MSH assay is specific for diAc-α-MSH, α-MSH and desAc-α-MSH, and crossreacts with ACTH at a low level (10%). Briefly, the standard diluent consisted of 0.01 M phosphate buffer (pH 7.4), 0.14 M NaCl, 0.025 M EDTA and 0.5% (w/v) bovine serum albumin (BSA). In each assay, the standard or sample was reacted with anti-α-MSH serum R24 (final dilution 1:21,000) for α-MSH assay and with anti-ACTH serum R22 (final dilution 1:18,000) for ACTH assay. After incubation for 24 hours, normal rabbit serum (final dilution × 50), goat anti-rabbit γ–globulin serum (final dilution 1:10) and 5% (w/v) polyethylene glycol 6000 (M.W. 7,500) were added to the reaction mixtures. After incubation for 2 hours, the mixtures were centrifuged at 2,500 g for 30 minutes. The supernatants were removed by aspiration, and radioactivity in the precipitate was counted with a gamma counter (ARC-100, Aloka, Japan). The RIA was performed at 4 °C.

### Gel filtration chromatography

Gel filtration was carried out on a Sephadex G-25 fine column (1.0 × 50 cm, GE Healthcare UK Ltd, England) or Sephadex G-75 fine column (1.0 × 100 cm, GE Healthcare UK Ltd, England) using 1 M acetic acid as an eluent. The eluate was collected in 0.5 ml (Sephadex G-25 column) or 1.0 ml (Sephadex G-75 column) volumes and the fractions were lyophilized. The lyophilized fractions were dissolved in the standard diluent for RIA. The column was calibrated with blue dextran (Kav 0), carbonic anhydrase (30 kDa), Lysozyme (14 kDa), ACTH (4.5 kDa), α-MSH (1.6 kDa), ACTH(1–8) (1.1 kDa) and dbcAMP (Kav 1.0).

### Analytical reverse-phase HPLC

Analytical reverse-phase HPLC separations were achieved using a SLC-6B high-performance liquid chromatograph system (Shimazu, Kyoto, Japan) equipped with a SPD-7A detector. The α-MSH-like immunoreactivity (α-MSH-LI) fraction obtained by gel filtration was loaded onto an ODS column (4.6 × 150 mm, Myghtysil RP-18 GP 5 μm, Kanto chemical Co. inc., Tokyo, Japan). The column was eluted with a linear gradient of CH_3_CN in 0.01 N HCl from 0% to 60% for 30 minutes at a flow rate of 1 ml/min. The fractionated samples were lyophilized, and then dissolved in the standard diluent for the radioimmunoassay.

### Mass spectrometric analysis

The α-MSH-LI fraction obtained by reverse-phase HPLC was applied to the target and mixed with saturated α-cyano-4-hydroxycinnamic acid (CHCA) solution. Mass spectrometry experiments were carried out on a Bruker Ultraflex TOF/TOF (Bruker Daltonics, Bremen, Germany). All searches were carried out using Flex Analysis software (Bruker Daltonics, Bremen, Germany) and post source decay (PSD) fragment ion spectra identified using the Mascot protein sequence database (Matrix science Inc. UK).

### Effect of ACTH(1–8) on melanin production and binding to MC-1R

Mouse melanoma cell line B16^43^, which express MC-1R and produce melanin, were purchased from Health Science Research Resources Bank (Osaka, Japan). Radioligand receptor binding studies on adhesive B16 cells were performed with minor modification to previous descriptions^44^. Briefly, B16 cells were cultured at a density of 1 × 10^5^ cells in a 24-well plate, washed by a binding buffer, 50 mM HEPES-KOH (pH 7.4) containing 120 mM NaCl, 2.5 mM KCl, 1.2 mM MgCl_2_, 15 mM NaAcO, 10 mM glucose and 0.2% BSA, and incubated for 6 h at 15 °C with [^125^I]-α-MSH (10,000  cpm) and the desired amount of unlabeled α-MSH or ACTH(1–8). Non-bound [^125^I]-α-MSH was aspirated and cell-bound [^125^I]-α-MSH was collected by 1 M potassium hydroxide and counted in a well-type gamma counter. Relative binding was calculated as the difference between [^125^I]-α-MSH bound in the absence (total binding, 100%) and presence of 1 μM α-MSH (non-specific binding, 0%) on B16 cells.

Melanin production activity was performed as follows: B16 cells and normal human melanocytes (TOYOBO. Osaka, Japan) were seeded at the density of 1 × 10^5^ cells in 24-well plates and allowed to attach overnight. Cells were treated with α-MSH or ACTH(1–8) at concentrations between 10^−7^–10^−11 ^M or 10^−6^–10^−11 ^M, respectively, and incubated for 72 hours. Cells were solubilized in 1 M NaOH at 60°C, and melanin content was measured spectrophotometrically at 475 nm. Melanin levels were expressed as 100% and 0% in the presence of 1 μM α-MSH and absence of α-MSH, respectively.

ASP(87–132)-NH_2_ and either 10^−8^ M α-MSH or 10^−7 ^M ACTH(1–8) were added to the B16 cells to compare melanin production via binding to MC-1R. Melanin levels were shown as 100% in the presence of 100 nM α-MSH or 1000 nM ACTH(1–8) and 0% in the absence of α-MSH or ACTH(1–8), respectively.

### Detection of trypsin-like enzyme activity by Boc-Phe-Ser-Arg-MCA cleavage assay

Trypsin-like enzyme activity in UV-irradiated auricles was determined using a Boc-Phe-Ser-Arg-MCA (Peptide Institute Inc., Osaka) cleavage assay as follows. Auricles were homogenized in 50 mM Tris-HCl buffer (pH 7.4) at 4 °C, and then centrifuged at 10,000 rpm, at 4 °C for 30 min. The supernatants were collected and stored at −20 °C. The auricle extracts were diluted in 50 mM Tris-HCl buffer (pH 7.4), and Boc-Phe-Ser-Arg-MCA (final concentration, 100 μM) was added to the solution, including the extracts. The solution was incubated at 37 °C for 2 hours, and the cleaved 7-amino-4-methyl coumarin was measured (excitation/emission, 355/460 nm).

### Determining protease molecular weight

We performed gel filtration on a CL-6B column (1.0 × 30 cm, GE Healthcare UK Ltd, England) using 20 mM Tris-HCl (pH 7.4) as an eluent. Trypsin-like protease activity in the fractionated samples (1 ml) was measured by Boc-Phe-Ser-Arg-MCA cleavage assay as described above. The column was calibrated with blue dextran (Kav 0), aldorase (160 kDa), bovine serum carbonic anhydrase (30 kDa), and dbcAMP (Kav 1.0).

### Western blot analysis

SDS-PAGE^45^ was performed using a Mini-Protean 3 Cell electrophoresis apparatus (Bio-Rad Laboratories, Hercules, CA). Auricle extracts were loaded onto a 15% (w/v) polyacrylamide gel. Proteins were then transferred to a nitrocellulose membrane (Protran BA85, GE Healthcare UK Ltd, England) through electrophoresis in a Mini Trans-Blot Cell 3 chamber (Bio-Rad Laboratories, Hercules, CA)^46^. Nitrocellulose membranes were blocked with 1% BSA. Blocked membranes were incubated with anti-ACTH rabbit antibody (R22), anti-plasminogen rabbit antibody (Assaypro LLC., St. Charles, MO USA) or anti-tryptase mouse monoclonal antibody (Thermo Fisher Scientific Anatomical Pathology, Fremont, USA) and then with horseradish peroxidase (HRP)-conjugated anti-rabbit IgG goat antibody (Biosource, Camarillo, CA) or HRP-conjugated anti-mouse IgG goat antibody (Biosource, Camarillo, CA). The protein bands were subsequently visualized by a chemiluminescent detection system^47^.

### Ethics Statement

All experimental protocols were approved by the ethics committee of the University of Shizuoka. This study was carried out in accordance with the guidelines of University of Shizuoka and of Nihon Pharmaceutical University.

## Additional Information

**How to cite this article**: Yamamoto, H. *et al.* Melanin production through novel processing of proopiomelanocortin in the extracellular compartment of the auricular skin of C57BL/6 mice after UV-irradiation. *Sci. Rep.* **5**, 14579; doi: 10.1038/srep14579 (2015).
